# Editorial: Exercise and its role in regulating energy metabolism—Insight from intramuscular mechanisms and cellular signaling

**DOI:** 10.3389/fphys.2023.1169923

**Published:** 2023-03-06

**Authors:** Kohei Kido, Rasmus Kjøbsted

**Affiliations:** ^1^ Faculty of Sports and Health Science, Fukuoka University, Fukuoka, Japan; ^2^ Institute for Physical Activity, Fukuoka University, Fukuoka, Japan; ^3^ August Krogh Section for Molecular Physiology, Department of Nutrition, Exercise, and Sports, Faculty of Science, University of Copenhagen, Copenhagen, Denmark

**Keywords:** exercise, energy metabolism, metabolic homeostasis, glucose uptake, fatty acid oxidation, skeletal muscle, molecular regulation, cellular signaling

Exercise is a powerful physiological modulator of metabolism that represents a cornerstone in the prevention and treatment of various metabolic diseases (e.g., obesity, type 2 diabetes, and sarcopenia). The protective effect of exercise on metabolic abnormalities involves multiple tissues, of which skeletal muscle is highly important because it is a major determinant of resting energy expenditure (Zurlo et al., 1990) that may increase more than 100-fold during vigorous exercise (Sahlin et al., 1998). It is well established that exercise enhances skeletal muscle energy utilization, protein turnover, and cytoskeleton remodeling (Hargreaves and Spriet, 2020). Over the past couple of decades much effort has been made in order to elucidate the mechanisms by which exercise alters whole-body and skeletal muscle metabolism. Many mechanisms have been ascribed to transcriptional, translational, post-translational, and even epigenetic modification but many more are still to be uncovered.

Most studies investigating mechanisms of metabolic adaptations focus on the overall response to exercise in a group of homogeneous subjects. Yet, recent observations suggest that much can be gained by also taking advantage of individuals who are less responsive or even non-responsive to exercise interventions. Hingst et al. (2022) showed that improvements in insulin-stimulated skeletal muscle glucose uptake following acute aerobic exercise are almost absent in some individuals and varies markedly across the spectrum of individuals from a group of homogeneous subjects (CV = 52%). In addition, low responders to the adaptation of aerobic capacity (VO_2max_) and muscle growth after aerobic and resistance exercise training are also known to exist (Vollaard et al., 2009; Roberts et al., 2018) but the underlying causes have not yet been fully elucidated. The heterogeneous exercise responses are likely caused by a complex interplay between extrinsic (environment) and intrinsic factors (e.g., genetics, gender, age and ethnicity). Research into these factors is important to understand why individuals benefit differently from exercise interventions that may ultimately help tailor exercise-based programs to optimize training adaptations as well as disease prevention strategies.

In this Research Topic, Gasser et al. have examined whether the angiotensin-converting enzyme gene variant (ACE-I/D) and fitness level associate with inter-individual differences in local and systemic aspects of metabolic efficacy during exercise. They propose that the ACE-D allele influences differences in acute exercise-induced aerobic metabolic processes at the level of skeletal muscle and lung tissue in different fitness states. They further argue that in aerobically fit subjects, dependence on mitochondrial function in working muscle is relatively more important for increased power output in D-allele carriers than in non-carriers.

In addition to genetic polymorphisms, differences in gender are one of the most significant factors influencing metabolic responses in mammals. Fortino et al. subjected young male and female individuals to eccentric contraction exercise in order to evaluate gender differences in the myogenic and inflammatory response. It has long been known that eccentric exercise increases satellite cell expression and inflammatory responses in human muscle (Peake et al., 2017; Nederveen et al., 2018) and now Fortino et al. are able to confirm that these responses are greater in male vs. female individuals. While there is no consensus on the effect of sex hormones on inflammation and regeneration for methodological reasons (Tiidus et al., 2005; Enns et al., 2008; Iqbal et al., 2008; Velders et al., 2012), Fortino et al. speculate that the observed sex-based differences may be explained by the higher concentration of testosterone and estrogens in male vs. female individuals, respectively.

In order to identify factors that regulate the inter-individual metabolic differences occurring following exercise, it is also important to develop unique experimental models that allow control of external and internal factors affecting the metabolic responses. By the use of omics analyses, Mengeste et al. describe RNA as well as intracellular and extracellular protein profiles after two types of electrical stimulation in primary human myotubes. Despite that these profiles resemble several features of exercise *in vivo*, not all changes seen after whole-body exercise could be reproduced in the myotubes. The authors ascribe these discrepancies to lack of circulating hormones and alterations in the microenvironment that occur during exercise *in vivo*. Because the two types of electrical stimuli are applied to myotubes differentiated from satellite cells of both young men and middle-aged women, the authors are able to stratify RNA and protein expression profiles based on age and/or gender. Furthermore, leukemia inhibitory factor (LIF) is identified as a myokine released from contracting muscle that enhances glucose uptake in cultured myotubes. This suggests that LIF plays a role in autocrine regulation of muscle glucose metabolism during exercise.

AMPK β is regarded as the glycogen-binding subunit of the AMPK heterotrimeric complex as it contains a carbohydrate-binding module (Polekhina et al., 2003). Introducing knock-in mutations in the glycogen-binding domains of AMPK β1 and β2 (DKI), Janzen et al. seek to explore the molecular mechanisms that regulate intramuscular glucose utilization during exercise. Janzen et al. identify that AMPK DKI mice have decreased exercise endurance and maximal running capacity likely due to higher and lower glucose and fat oxidation during exercise, respectively. Although the knock-in mutation in the glycogen-binding region of AMPK β does not affect glycogen content in rested and exercised muscle, findings presented by Janzen et al. suggest that the binding of AMPK β to glycogen is necessary to preserve exercise endurance and maximal running capacity by securing proper substrate utilization during exercise.

In summary, this Research Topic has advanced our understanding of the mechanisms regulating whole-body and skeletal muscle metabolism in response to various forms of exercise. Specifically, the series of articles have addressed some of the factors that cause inter-individual differences in metabolic changes following exercise. Moreover, new regulatory mechanisms of energy metabolism at rest and during exercise have been elucidated *via* unique physiological and biological approaches. Such findings are ultimately important for establishing effective, efficient, and personalized exercise programs for all individuals in order to secure a healthy lifestyle and prevent development of metabolic diseases.

Exercise adaptations are controlled by numerous regulatory and interacting protein networks of which many still need to be fully described. In addition, non-protein molecules such as lipids and metabolites are presumably also mediators of exercise adaptive responses but have not been adequately studied in detail. Mechanistic investigations of these molecular determinants will provide the basis for new hypotheses and ideas about the mechanisms responsible for the exercise-induced adaptations in skeletal muscle. This will contribute to our understanding of the health beneficial and therapeutic effect of regular exercise that may be exploited for pharmacotherapies to combat various lifestyle and chronic diseases.
